# Conversion of wood-biopolymers into macrofibers with tunable surface energy via dry-jet wet-spinning

**DOI:** 10.1007/s10570-018-1902-4

**Published:** 2018-06-19

**Authors:** Tiina Nypelö, Shirin Asaadi, Günther Kneidinger, Herbert Sixta, Johannes Konnerth

**Affiliations:** 1 Division of Applied Chemistry, Department of Chemistry and Chemical Engineering, Chalmers University of Technology, Gothenburg, Sweden; 2 Department of Material Sciences and Process Engineering, Institute of Wood Technology and Renewable Materials, University of Natural Resources and Life Sciences, Vienna, Austria; 3 Department of Bioproducts and Biosystems, Aalto University, Espoo, Finland

**Keywords:** Regenerated fibers, Wood-biopolymers, Forest biomaterials, Fiber surface energy, Adhesion force mapping

## Abstract

**Abstract:**

Surface chemistry of regenerated all-wood-biopolymer fibers that are fine-tuned by composition of cellulose, lignin and xylan is elucidated via revealing their surface energy and adhesion. Xylan additive resulted in thin fibers and decreased surface energy of the fiber outer surfaces compared to the cellulose fibers, or when lignin was used as an additive. Lignin increased the water contact angle on the fiber surface and decreased adhesion force between the fiber cross section and a hydrophilic probe, confirming that lignin reduced fiber surface affinity to water. Lignin and xylan enabled fiber decoration with charged groups that could tune the adhesion force between the fiber and an AFM probe. The fibers swelled in water: the neat cellulose fiber cross section area increased 9.2%, the fibers with lignin as the main additive 9.1%, with xylan 6.8%, and the 3-component fibers 5.5%. This indicates that dimensional stability in elevated humidity is improved in the case of 3-component fiber compared to 2-component fibers. Xylan or lignin as an additive neither improved strength nor elongation at break. However, improved deformability was achieved when all the three components were incorporated into the fibers.

**Graphical Abstract:**

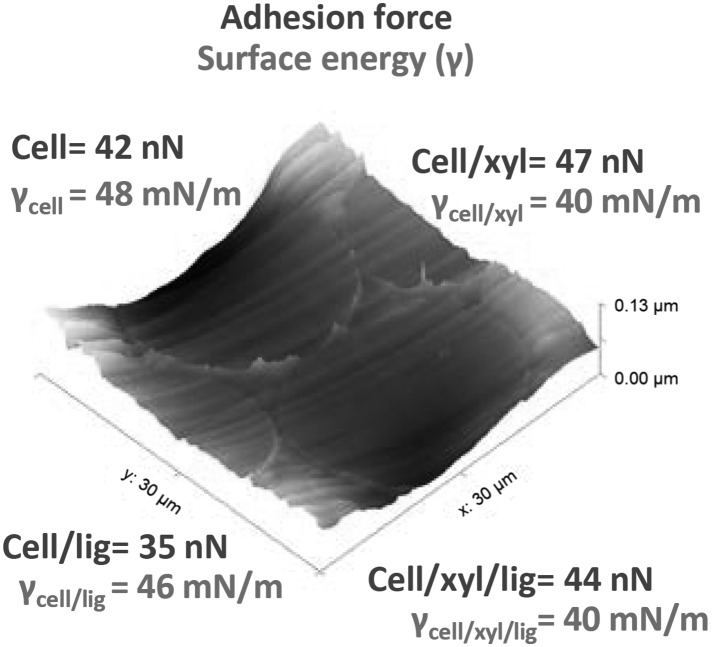

**Electronic supplementary material:**

The online version of this article (10.1007/s10570-018-1902-4) contains supplementary material, which is available to authorized users.

## Introduction

Ionic liquids enable simpler, less-solvent consuming regenerated cellulose fiber spinning process compared to the traditional ones where cellulose is dissolved in N-methylmorpholine-N-oxide (NMMO) or recrystallized via mercerization in alkaline conditions (Adanur 1995). A processing route for cellulose that involves ionic liquids, salts with a melting point lower than 100 °C, has been suggested and has exploded the fiber manufacturing horizon (Michud et al. 2016; Kosan et al. 2008). The approach is simpler than the traditional ones with the promise of reduced water and solvent consumption. The remarkable dissolving power further opens opportunities for fiber spinning from lower quality raw material than possible now (Ma et al. 2016).

Wood is a viable source for cellulose for high volume applications such as textile fibers mainly due to its abundance and being of a non-food source. Alongside with cellulose, wood comprises of hemicelluloses, lignin and extractives. Consequently, spinning from ionic liquid dopes is not only interesting for cellulose but also lignin containing fibers have been demonstrated from dissolved lignin containing biomass (Sun et al. 2011). Lignin is envisioned as a fiber surface chemistry modifier (Gindl-Altmutter et al. 2014) and carbon fiber precursor (Thunga et al. 2014). Lignin containing fibers have been suggested as naturally dyed textile fibers (Ma et al. 2015).

Hemicelluloses in fibers have been linked as regulators for cellulose fibril aggregation in Kraft pulping hence leading to increased stability (Hult et al. 2001). Deliberate use of hemicelluloses in wet spinning process of wood-based materials in ionic liquids is yet to be established in industrial scale. Schildt and Liftinger (2014) showed that incorporation of 7.5% of xylan into viscose fibers increased water retention and maintained strength. Singh and Murthy (2017) reported reduced fiber–fiber friction of viscose fibers with the addition of xylan which was concluded to be due to the circular cross section that resulted from hemicellulose addition. In the case of native wood-based cellulose fibers, hemicellulose residues are present and affect the association between cellulosic units (Hult et al. 2001) and in some cases have a tremendous effect on processability and yield (Chaker et al. 2013). Based on studies on nanocellulose, there is an expectation that hemicelluloses in cellulose matrix contribute to grease resistance of final products or act as friction modifiers (Kisonen et al. 2015).

Bulk of materials determines performance such as strength and deformation. However, surface properties are dominant when it comes to adhesion, bonding with other materials, friction characteristics and functionality. While preparation and strength of regenerated fibers is increasingly investigated, the surface characteristics have been less in the scope. We spun wood-biopolymer based fibers according to the Ioncell-F process (Michud et al. 2016) using cellulose, xylan, lignin and their mixtures dissolved in 1,5-diazabicyclo[4.3.0]non-5-enium acetate (DBNHOAc). Varying the composition of the fibers was aimed on one hand for adjusting the polarity of the fiber surface and on the other hand the mechanical properties. The latter should additionally be able to be addressed by changing the draw ratio (DR). The role of lignin and xylan was to regulate the acid and base potential of the fibers. Lignin is expected to affect the polarity of the fiber surfaces, while xylan was targeted to increase the acid component of the surface energy of the fibers to provide an additional parameter for tuning the fiber surface properties. We apply the Wilhelmy plate approach to determine the surface energy components of the fibers (Miller and Young 1975; Miller et al. 1983). We have modified the traditional single fiber and the Wilhelmy plate test into a measurement involving a series of single fibers in a Wilhelmy plate assembly. We provide additionally a unique exploration of fiber cross section surface properties and compare those to the properties of the fiber outer surface. The limited area of the cross section is not accessible via the standard wetting method and the analysis was executed by Atomic Force Microscopy (AFM) adhesion force measurements in air. This enabled high resolution observation of the fiber cross section morphology to identify possible phase separation or compositional gradient. The fiber characteristics, strength, wetting, swelling, outer and inner fiber surface chemistry could be tuned by adjusting the composition. These findings are the first to elucidate the surface energy of wood-biopolymer fibers regenerated from ionic liquids and are directly relevant to the current interest in biomaterials science to prepare all-wood-biopolymer fibers as well as to develop fiber spinning based on ionic liquid dopes.

## Results and discussion

### Surface properties of the fibers

**Fig. 1:**
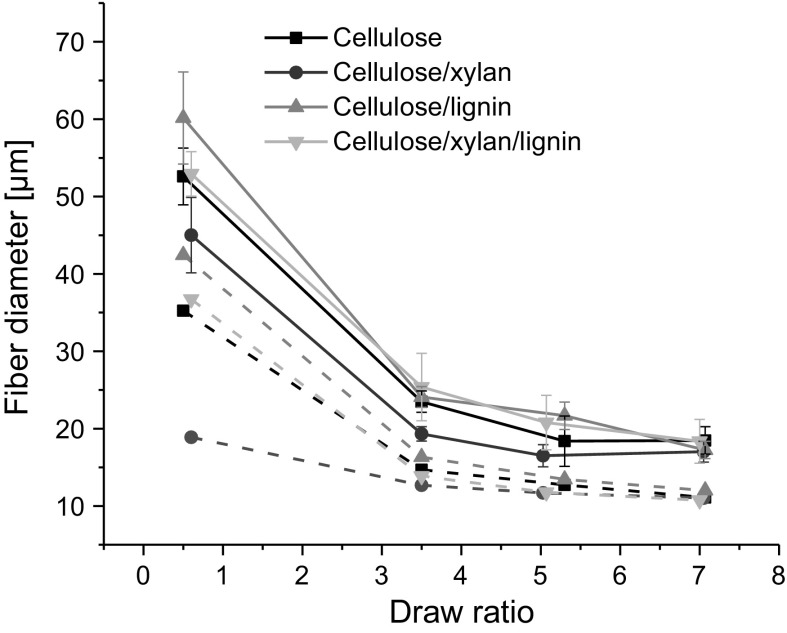
Fiber diameter determined by light microscopy (solid lines) and from vibroscope measurements (dashed lines)

Cellulose, cellulose/xylan, cellulose/lignin and cellulose/xylan/lignin dopes were successfully drawn into fibers with diameter decreasing with increasing draw ratio (Fig. 1a). The lowest draw ratios resulted in largest fiber diameters, between 45 and 60 µm, the cellulose/xylan fibers being the thinnest and the cellulose/lignin the thickest ones. The highest draw ratio resulted in lesser deviation in fiber diameters, all of them being between 17 and 18.5 µm.

The fiber diameter relation to draw ratio determined by light microscope and the experimental titer for the cellulose and cellulose/lignin fibers followed the same trend but were of different magnitude: the fiber diameters determined by light microscope were larger compared to the ones calculated from the experimental titer (solid vs. dashed lines in Fig. 1a). Applying an empirical correction factor to account for changes due to drying and conditioning after spinning balanced the difference for cellulose and cellulose/lignin fibers (Figure S1). However, a simple correction factor did not improve the fit of the two diameters in the case of the xylan containing fibers so that cellulose/xylan diameter deviated in the low DR range and cellulose/xylan/lignin in the high DR range. Hence, in the case of xylan this discrepancy is of other than of calculation origin and likely due to molecular orientation/packing different from that of the other components, or due to frictional aspects during spinning caused by xylan.

Water, diidomethane, ethylene glycol and glycerol contact angle on the fiber surface was determined using the Wilhelmy plate method (Online Resource Figure S2). A Washburn sorption method is suggested to be superior for fibers due to their evident liquid uptake and capillary action (Stuart et al. 2015). However, measurements with this mode resulted in no apparent sorption taking place and hence the Wilhelmy plate method was used. The fibers were uniform and the surface smooth (Online Resource Figure S3). Hence, the potential artifact of fiber roughness on the contact angle (Heng et al. 2007) is negligible.

Water contact angle for cellulose fibers was determined to be zero indicating full wetting. The contact angle of pure cellulose has been reported to be low (14°) compared to that of more complex cellulosic fibers (23–65°) (Hodgson and Berg 1988). The water contact angle for the fibers here increased with the lignin concentration in the fibers. This is expected due to commonly accepted lower polarity of lignin that is due to the aromatic backbone and phenolic hydroxyl groups (Yang and Pan 2015), while cellulose is abundant with primary and secondary hydroxyl groups on the sugar backbone. Contact angle of the other polar liquid used, ethylene glycol and the apolar diidomethane are also presented in Online Resource Figure S2. These values were further used for calculating the surface energy and the acid and base components to identify surface characteristics of the fibers.

**Fig. 2:**
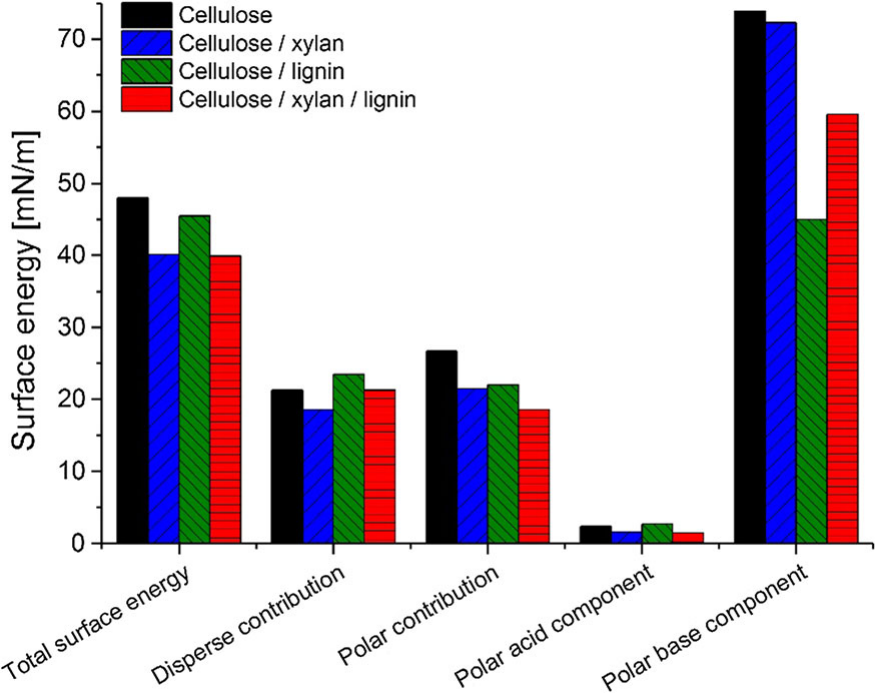
Total surface energy, its disperse and polar contributions, and the acid and base components of the polar contribution of the fibers calculated based on the acid–base theory

All the fibers were low surface energy materials of less than 50 mN/m (Fig. 2). The total surface energy of the fibers decreased when lignin and/or xylan was added being more pronounced with the addition of xylan. The surface energy decrease with the lignin addition is expected since lignin is considered as the hydrophobic component in the wood cell matrix. The disperse part of the surface energy increased with addition of lignin whereas the polar contribution in the surface energy decreased. Addition of xylan decreased the disperse and polar contribution. The magnitude of the decrease in surface energy with xylan is surprising considering its mainly hydrophilic structure. The polar contribution of the 3-component fibers decreased the most which is probably due to the decrease in cellulose content together with incorporation of lignin. Surface energy of natural fibers with varying composition has been reported to correlate with the amount of cellulose (Blatazar-y-Jimenez and Bismarck 2007).

Based on the determination of the acid and base components, the fibers were of pronounced base-character (Fig. 2). Addition of lignin to the cellulose matrix decreased the polar base component significantly and increased the acid component whereas the effect of xylan addition on this was minor. It has been reported that the basicity of natural fibers increases with increasing cellulose content (Heng et al. 2007). For cellulose, the basicity has been explained by the hydroxyl groups being occupied in intra and inter molecular hydrogen bonding leaving the ether linkages free to contribute to the basicity (Heng et al. 2007). Rojo et al. (2015) have shown that increasing lignin amount on cellulose nanopapers decreases the base component and results in amphoteric character of the papers. Here it was noted that the effect of lignin was more pronounced to the base component than the cellulose content: proton acceptor capability of the fibers containing the highest amount of lignin decreased while donor capability increased. This amphoteric character could be potential for improving filler–polymer compatibility in composites. Surprisingly the acid component of the xylan containing fibers did not increase although one would assume this based on the presence of glucuronic acid in the xylan additive. Clearly the polarity and surface chemistry of the fibers could be tuned by varying their composition.

The cellulose fiber cross sectional area increased in water 9.2% (Table 1). The swelling reduced to 6.5% with the thinner fibers (diameter decreased from 53 to 19 µm with increase of the DR). Microscope images of the fibers confirm that immersion to water affects the fiber morphology seen as bending of the fibers (Online Resource Figure S3). The lignin containing cellulose fibers swelled almost the same extent (9.1%) as the neat cellulose fiber while being slightly thicker, 60 µm. This is surprising as one would expect lignin to reduce the water uptake due to being a less hydrophilic molecule than cellulose. However, swelling is not only a matter of hydrophilicity but of structure as well. The xylan containing and the 3-component fibers swelled significantly less, approx. 6.2 and 5.5%, respectively, than the cellulose ones. It might be that the presence of xylan enhances the packing of the molecules, hence reducing the penetration of water inside the fibrillar structure.

**Table 1:** Change in fiber cross section area in water

Fiber composition	Cross sectional area change
Cellulose	9.19% ± 4.57
Cellulose/xylan	6.20% ± 2.87
Cellulose/lignin	9.05% ± 2.86
Cellulose/xylan/lignin	5.52% ± 3.70
Cellulose (DR7)	6.47% ± 4.24

The fiber perimeter is one of the parameters that define contact angle of liquids in the Wilhelmy plate method (Eq. 3 in Experimental). Since the fibers were noted to swell in water, we estimated the error that this causes to the contact angle data and consequently to the surface energy. The determination of extent of swelling was carried out by 2 min exposure, while the contact angle was measured within 20 s immersion. Since we do not know the exact rate of swelling during the contact angle determination, the maximum swelling reported in Table 1 was used for the calculations. The experimental contact angle was used as the contact angle for the dimension before swelling. Equation 1 was then used to estimate the contact angle after the swelling. These water contact angle values were then used for the acid base modeling of the total surface energy and converted into a difference in percentage (Online Resource Table S1). The change was the highest, 5%, for the cellulose/lignin fibers and less than 0.6% for the others. Only the effect on the water contact angle was considered since no significant swelling was noticed in diidomethane or ethylene glycol.

**Fig. 3:**
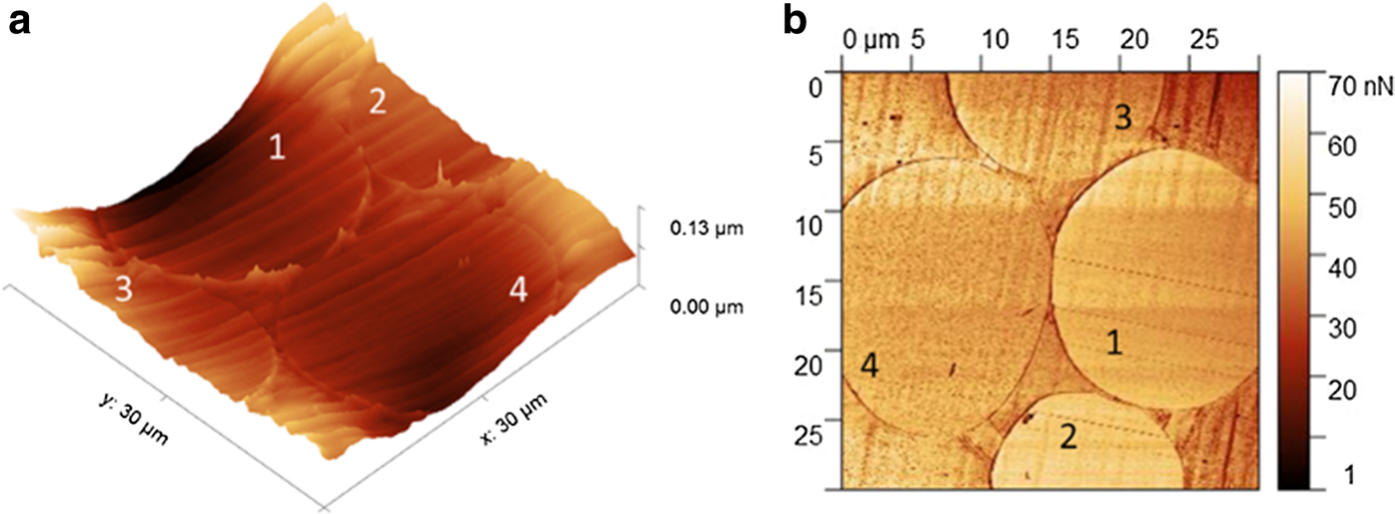
AFM height (**a**) and adhesion force scan (**b**) of the cellulose (1), cellulose/xylan (2), cellulose/lignin (3) and cellulose/xylan/lignin (4) fibers embedded in PLA

The shape of cellulose fiber cross sections spun from ionic liquid dopes have been reported to be round (Hummel et al. 2015) complying to the shape of the traditional regenerated fibers spun from NMMO (Fink et al. 2001; Chanzy et al. 1990). This was the case also with the fibers spun here, and additionally, AFM height imaging revealed similar appearance and roughness among the fiber cross sections (Fig. 3a).

Adhesion between two surfaces is related to their surface energies. Consequently, it has been suggested that the AFM adhesion force measurements can be related to wetting by relation of adhesion force (F_A_) to the contact angle,

1 $${\text{F}}_{{\text{A}}} = {\text{x}}{\uppi }{\text{R}}_{{\text{P}}} {\text{W}}_{{\text{A}}}$$

where R_P_ is the radius of the spherical AFM tip, x depends on the tip properties, and,

2 $${\text{W}}_{\text{A}} =\upgamma\left( {{ \cos }\uptheta + 1} \right)$$

Pietak et al. (2007) have established the AFM adhesion force measurement in conjunction with wetting characterization for lignocellulosic fibers before and after treatments including steam explosion, enzyme treatment and acetylation. Using a calibration on smooth substrates with varying surface energy, they showed that there is a relation between the contact angles of these fibers and the adhesion force.

We explored the adhesion force of the fiber cross sections to a standard silicon AFM tip in ambient environment similar to the approach recently shown by Colson et al. (2017). While the contact angle measurements performed by dipping fibers into the polar and apolar liquids are to probe the fiber outer surface, the adhesion force mapping were performed on the internal part of the fiber, the cross section.

Distribution of the molecules inside regenerated blend cellulose fiber structures have so far not been reported. It has been previously shown that adhesion force mapping can be used to identify polysaccharides from one another in films at corresponding high resolution (Nypelö et al. 2017). The adhesion force scanning contrast is presented in Fig. 3b where the lighter color represents higher adhesion force values (fibers 1 and 2) and darker represents lower values (fibers 3 and 4). The rather homogenous adhesion value distribution on a fiber further implies a homogenous cross section structure and no core–shell organization for the fibers presented in this work. Adhesion force values for each of the frames (presented in Online Resource Table S1) followed the trend: cellulose and cellulose/xylan/lignin fibers had similar adhesion to the tip, the lignin containing fibers had the lowest affinity and the xylan containing the highest adhesion towards the polar tip. The adhesion force values may not be regarded quantitative, but allow relative comparison within one frame.

**Fig. 4:**
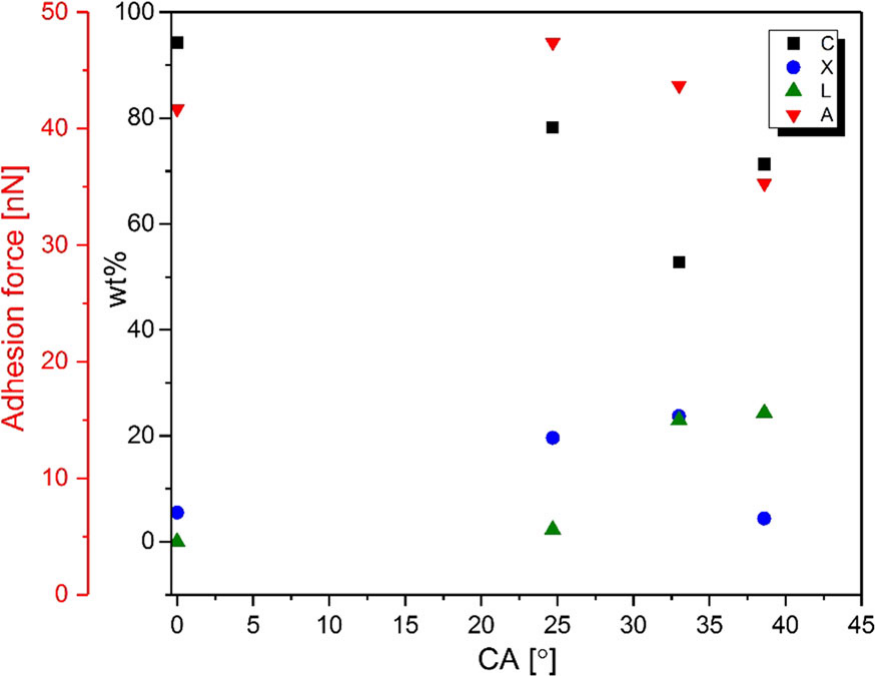
Water contact angle (CA) of the fibers contrasted to their composition (black y-axis) and cross section adhesion force (red y-axis). For the composition scale C denotes cellulose (black squares), X xylan (blue squares), L lignin (green squares). The red circles are for adhesion force (A) of the cellulose, cellulose/xylan, cellulose/xylan/lignin and cellulose/lignin from left to right. (Color figure online)

The source of the adhesion in the AFM measurement was considered to be mainly due to polarity resulting from affinity of the fiber surface to the hydrophilic silicon tip. The adhesion force values should reflect the polarity difference of the fibers since adhesion is related to the water contact angle (Eqs. 1 and 2). To further elucidate the relationship of the polarity to the composition we have combined the water contact angle (Figure S2), the fiber cellulose, xylan or lignin content (Table 2) and the measured adhesion force of the fiber cross section (Table S1) in Fig. 4. The adhesion force was related to the water contact angle (see red downward triangles in Fig. 4) except in the case of the fiber with the highest cellulose content (the cellulose fiber with no additives). Based on the chemical properties of cellulose and hemicelluloses one would expect similar adhesion to the tip. However, the adhesion of xylan containing fibers was higher than that of the neat cellulose. The same was true for the 3-component fiber. This implies that there is contribution for other source than polarity that dominates the tip-fiber interaction. It is clear that an increase in lignin content (green upward triangles in Fig. 4) increases the water contact angle. Xylan or cellulose content (blue spheres and black squares) did not have a linear influence on the fiber wetting by water.

**Table 2:** Fiber composition by weight

Components	Cellulose (wt%)	Cellulose/xylan (wt%)	Cellulose/lignin (wt%)	Cellulose/xylan/lignin
Cellulose	94.24	78.19	71.33	52.75
Xylan	5.52	19.56	4.35	23.66
Lignin	0.00	2.25	24.31	22.91
Cellulose: additive	94:6	78:22	71:29	53:47

### Mechanical response of the fibers

**Fig. 5:**
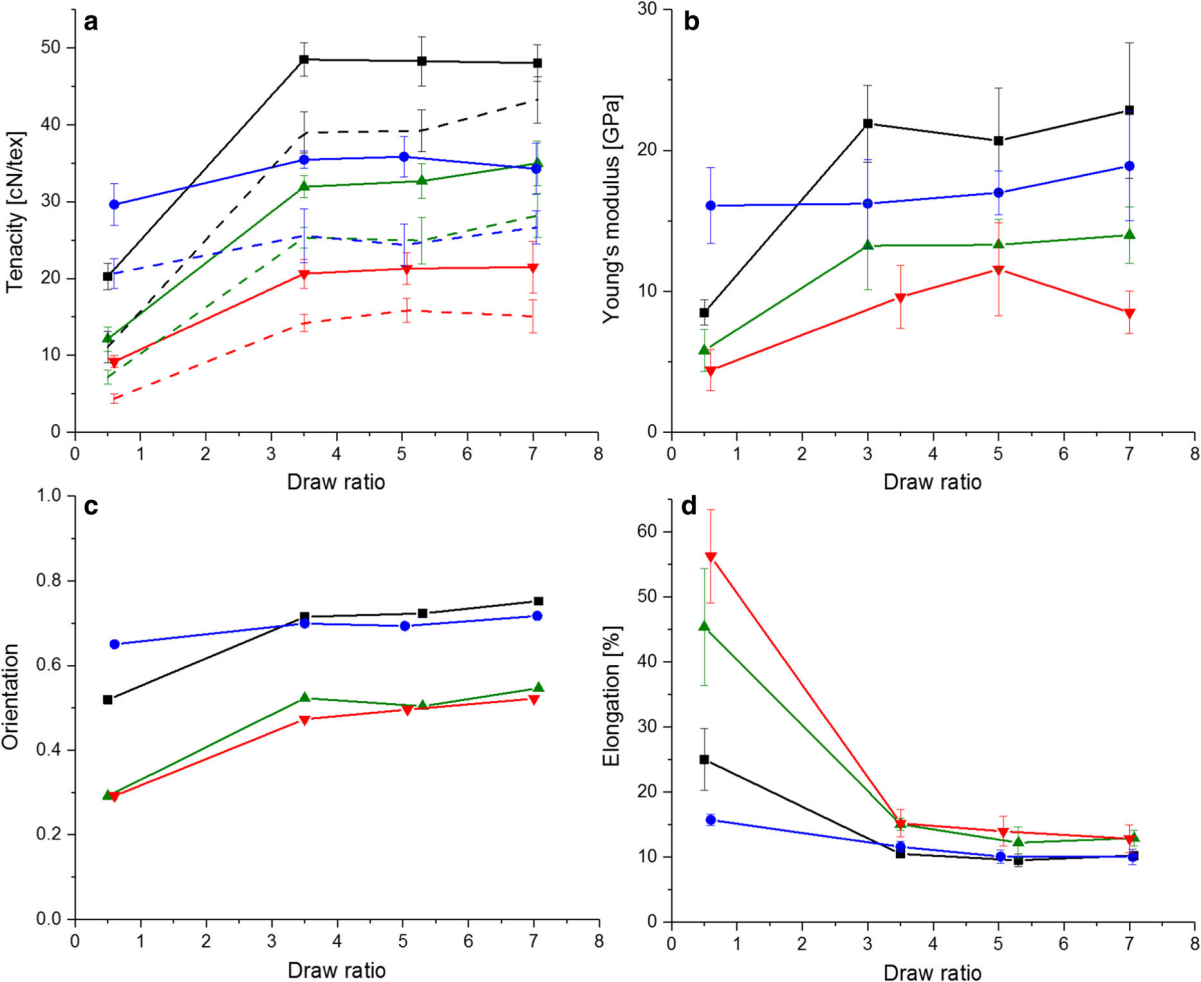
**a** Fiber tenacity (wet fiber data is presented with dashed lines, see color coding in electronic version), **b** Young’s modulus, **c** orientation and **d** elongation with respect to draw ratio for cellulose (black square), cellulose/xylan (blue circle), cellulose/lignin (green upward triangle), and cellulose/xylan/lignin (red downward triangle) fibers. (Color figure online)

The composition of the fibers is presented in Table 2. The cellulose content decreased from 94% to 78, 71 and 53% in the order of cellulose, cellulose/xylan, cellulose/lignin, cellulose/xylan/lignin. Cellulose fibers drawn with high draw ratio (thinnest) had a tenacity of up to 48 cN/tex (Fig. 5a) and are therefore the stiffest and strongest fiber type in both wet and dry condition. The strength reducing effect of lowered cellulose content has been reported (Sun et al. 2011) and is attributed to a decrease in the orientation of the fibers (Ma et al. 2015). All fiber types showed an expressed yielding point in dry condition (Figure S4) which disappeared when fibers were tested wet. As a trend, both Young’s modulus and tenacity are clearly affected by the ambient condition type (wet, conditioned). In contrast, within the draw ratios tested, elongation remained almost similar when tested in dry or wet condition (Figure S4).

Among the tested fibers, the cellulose/xylan fibers showed the highest tenacity and Young’s modulus (Fig. 5b), and the highest degree of orientation at the lowest applied DR (Fig. 5c). However, the orientation value for the low DR should not be emphasized in the analysis due to possible inaccuracies in the draw adjustment with low draw ratios. Hence, the finding needs further attention and testing to elucidate the role of xylan to fiber orientation and mechanical performance. The orientation of the other fibers first increased up to DR 3.5 after which the increase rate decreased (Fig. 5b). Despite the continuous increase in orientation, the 3-component fibers showed a remarkable loss in modulus at the highest draw ratio. This complies to the findings of Ma et al. (2015) who prepared fibers from cellulose-lignin blends and concluded that after certain addition the incorporation of lignin impedes the formation of a homogeneously ordered structure and that causes a decrease in the fiber tenacity and an increase in the elongation. Elongation of the fibers was the highest with low draw ratios, which may be considered of plastic deformation type (Fig. 5d). Cellulose and its orientation provides strength to the fibers, whereas the addition of lignin improved elongation (Fig. 5d). Highest deformability measured as elongation resulted when the additive lignin was combined with xylan.

## Conclusions

Lignin, the aromatic wood cell wall component, increased the water contact angle of the fiber outer surface and decreased adhesion of the internal surface (cross section) to a hydrophilic probe suggesting modification of the fiber surface. Lignin also reduced the base character of the fibers owing to higher fraction of acidic groups compared to the other components. Xylan was identified to be an efficient modifier for surface energy and decreased the total surface energy. Xylan additive in the fiber increased adhesion force between a hydrophilic probe and the fiber which implies that there are additional interactions spanning from molecular to morphological features that contribute to fiber surface adhesion. Interestingly, using the three components in the fibers resulted in reduced surface energy, increased strain and decreased swelling when exposed to water. The latter ones will be valuable regarding fiber stability in further uses. The former one is a fundamental base for further elucidation of the influence of the tunable fiber surface polarity to practical applications such as fiber-matrix adhesion. As expected, the long-chain cellulose molecules delivered strength in the fibers that were spun by dry-jet wet spinning from dopes containing cellulose, xylan and lignin in varying composition in an ionic liquid.

## Experimental section

### Materials

The cellulose was from Enocell birch prehydrolysis kraft pulp (476 ml g^−1^, Mw = 274.3 kg mol^−1^, Mn = 68.2 kg mol^−1^, PDI = 4) from Stora Enso, Finland, containing 93.25 wt% cellulose, 6.27 wt% hemicellulose and 0.48 wt% lignin. Xylan grade was the same as reported in Alekhina et al. (2014) isolated by cold caustic extraction from a birch Kraft pulp. It contained 1 mol% glucose, 96.7 mol% xylose, 0.5 mol% mannose, 0.3 mol% arabinose and 1.5 mol% 4-O-Methyl glucuronic acid. The weight average molecular mass of the xylan was 30,600 g mol^−1^ as determined by GPC measurement. The lignin was a beechwood organosolv lignin used also in Ma et al. (2015) containing 90.2 wt% lignin, 9.6 wt% hemicelluloses and 0.2 wt% cellulose. The lignin originates from the Fraunhofer Institute CBP, Germany. The 1,5-Diazabicyclo(4.3.0)non-5-ene (DBN) (99%, Fluorochem, UK) and glacial acetic acid (Merck, Germany) were used as received. (DBNH)OAc ionic liquid was synthesized by slowly adding equimolar amounts of acetic acid to DBN under cooling in a 2000 cc reactor at 80 °C (Michud et al. 2016; Parviainen et al. 2015). The reaction is exothermic, therefore synthesis was done under external cooling to keep the temperature constant.

### Preparation of spinning dopes

Targeted compositions of fibers were 100% cellulose, 75% cellulose/25% xylan, 75% cellulose/25% lignin and 50% cellulose/25% xylan/25% lignin. For preparing the spinning dopes the air-dried cellulose, lignin and xylan were dissolved in the ionic liquid under reduced pressure of 10 mbar and heating in a vertical kneader rotating at 30 rpm, between 80 and 85 °C, for 90 to 120 min. Subsequently, it was press-filtered through a layered filter mesh (GKD Ymax2, 5 µm nominal, Gebr. Kufferath AG, Germany) under 2 MPa. Cellulose and cellulose/xylan dopes prepared with 13 wt% concentration and cellulose/lignin and cellulose/xylan/lignin in 15 wt%. A homogenous air-bubble free dope was ensured by observing the dope with optical microscope.

### Spinning

Fibres were spun by a customized laboratory piston spinning system (Fourne´ Polymertechnik, Germany) described in Sixta et al. (2015) and draw ratios (DR) of approximately 0.5, 3, 5 and 7. The cylinder was loaded with the spinning dope, which was heated to a preset temperature to form a homogeneous, air bubble free spin dope. The solution was then extruded through a 200-hole spinneret, each with a capillary diameter of 100 µm and capillary length of 20 µm comprising to an L/D-ratio of 0.2. The spinning temperature was selected according to the rheology measurements (see Online Resource for the experimental description) and was in the range of 60–70 °C except for cellulose/xylan/lignin solution which was 95 °C. The dope (300–500 g) was spun via an air gap into a cold-water bath (10–15 °C), where the formed filament was led over a PTFE guide roller (at 20 cm depth) and via another guide onto a motor-driven godet couple. The rate of extrusion (Ve) was kept constant while the take-up velocity (Vtu) of the godet couple was varied according to the targeted DRs (Vtu/Ve).

The filaments were collected on the godet, carefully removed with a razor blade, washed first in cold (5 °C) and then with hot water (70 °C), air-dried and stored under controlled conditions (20 °C, 65% RH) for tenacity measurements.

### Fiber chemical composition

The chemical composition (carbohydrate, Klason lignin and acid-soluble lignin) of the fibers was analyzed according to NREL standard/TP-510-42,618 and the summary is presented in Table 2. The amount of carbohydrates was detected by high-performance anion exchange chromatography with pulse amperometric detection (HPAEC-PAD) using a Dionex ICS-300 system. The samples were prepared from cut fibers that were treated in 72% sulfuric acid at 30 ± 0.5 °C for 60 min. Then the hydrolysis solutions were diluted to 4% concentration and autoclaved for 1 h at 120 °C. After cooling, the samples were ready for analysis of the carbohydrates. The carbohydrates in the hydrolysates were quantified using a Dionex ICS 3000 HPAEC-PAD (Dionex, Sunnyvale, CA, USA) (Sixta et al. 2001). The method was adapted to the CarboPac PA20 column (Dionex, Sunnyvale, CA, USA). The content of MeGlcA was determined by acid methanolysis/GC (Sundberg et al. 1996). The cellulose and hemicellulose contents were calculated according to the amount of monosaccharides following the Janson formula (Janson 1970). For determining the insoluble lignin content, the autoclaved hydrolysis solutions were vacuum filtered through weighed filtering crucibles while the filtrate in a filtering flask was captured to determine acid soluble lignin. The crucible was dried with acid insoluble residue at 105 ± 3 °C. The acid-soluble lignin (ASL) was determined using a Shimadzu UV 2550 spectrophotometer at a wavelength of 205 nm and an absorption coefficient of 110 L g^−1^cm^−1^. The fibers are referred to as cellulose, cellulose/lignin, cellulose/xylan and cellulose/xylan/lignin fibers based on their main composition (Table 2).

### Mechanical and physical properties of the fibers

The linear density (titer) was determined with a vibroscope (Vibroskop 400, Lenzing Instruments GmbH & Co KG, Austria) with at least 50 mg dtex^−1^ pretension. The titer was measured three times permitting 1.5% variation to exclude artefacts. Tenacity and elongation (wet and conditioned in 23 °C, 50% relative humidity) were determined using a Vibrodyn 400 (Lenzing Instruments GmbH & Co KG, Austria). Ten fibres from each kind and draw ratio were tested at 20 °C and 65% RH. The gauge length was 20 mm and speed 20 mm min^−1^ according to DIN 53816. Fiber diameter was calculated from the titer determined by the vibroscope using densities of 1.5, 1.45 and 1.26 g cm^−3^ for cellulose, xylan and lignin. A density of the fibers was then estimated based on the composition presented in Table 2.

The fiber diameters were additionally determined with Zeiss (Germany) Axiocam 2 light microscope coupled with Zeiss Fluoarc fluorescence source and Zeiss Axiovision 4.8 software by measuring diameter of at least 10 fibers of each fiber set. Swelling of the fibers was determined by assembling the fibers on a holder and immersing them for 2 min in water. The diameter of each of the measured fibers was determined after the immersion and used for calculating cross section area assuming circle profile. The thickest fibers corresponding to the lowest set of draw ratio of each composition were used for evaluating the swelling if not stated otherwise.

### Birefringence

The average total orientation of each fibre sample was measured via its birefringence using a polarized light microscope. For the optical measurement, the filaments were mounted between two pieces of double-sided tape on a microscope slide. Three selected filaments from each sample were analyzed by means of a Zeiss Axio Scope A1 polarized light microscope equipped with a Leica B 5λ-Berek tilting compensator as described previously (Asaadi et al. 2016) The optical retardation was determined in triplicate from a selected spot along the fibre. Birefringence is defined as the retardation divided by the diameter; a birefringence of 0.062 was assumed to be equivalent to 100% orientation (Adusumalli et al. 2009; Lenz et al. 1994).

### Contact angle and surface energy determination

The contact angle of the thickest fibers of each composition with water, diidomethane, ethylene glycol, glycerol and formamide were determined using Krüss K100 force-tensiometer (Germany). For each measurement 10 individual fibers were distributed on a 12-mm holder and cut to length of 3 mm. The contact angles were determined using the Wilhelmy plate method:

3 $$\upsigma = {\text{F}}/\left( {{\text{L}}*{ \cos }\uptheta} \right)$$

where σ denotes interfacial tension, F the force experienced when the plate touches a liquid surface, L the sample perimeter (fiber diameters determined by light microscopy) and θ the contact angle. It is based on observing the pull exerted to the solid by the liquid it is being immersed in Miller and Young (1975), Miller et al. (1983). The receding and advancing contact angles are resolved by linear regression from the force versus location plot. The location being the fiber immersion depth to the liquid. The aim of the tensiometer measurements was to reveal the surface energy of the fibers. This is not accessible directly but can be calculated based on modeling on the contact angle data. We further wanted to reveal the disperse and polar components and for the latter the acid and base components. Hence, surface energy components were modeled with the acid–base model (Good and van Oss 1992; Good 1993; van Oss et al. 1988), using the contact angle values of water, diidomethane, ethylene glycol, and Krüss software Laboratory Desktop version 3.2.2.3068. The surface tension parameters of all the liquids used are presented in Table 3. For estimating the effect of change in the fiber perimeter due to swelling on the surface energy was done using a program Surface Energy V1.xls (Copyright © 2007 By Gerhard Sinn, BOKU University of Natural Resources and Life Science). It is a non-commercial program with the acid–base model embedded. It was used instead of the Krüss software only for evaluating the error caused by the change in the diameter because recalculation with new parameters was not possible with the commercial software.

**Table 3:** Liquids, their surface tension, and its components used for the surface energy determination. (*Source*: Krüss software Laboratory Desktop version 3.2.2.3068 database)

Liquid	Surface tension (mN/m)	Disperse component	Polar component	Acid component
Water	72.80	21.80	51.00	25.50
Diiodomethane	50.80	50.80	0.00	0.00
Ethylene glycol	48.00	29.00	19.00	1.92
Formamide	58.00	39.00	19.00	2.28
Glycerol	64.00	34.00	30.00	3.90

### Atomic force microscopy analysis

Fiber cross sections were imaged with Bruker Dimensions Icon AFM in air using PeakForce Quantitative Nanomechanical Mapping (QNM) mode and ScanAsyst Air (Bruker) tips with resonance frequencies of 70 kHz and spring constant of 0.4 N m^−1^. Prior to measurements the actual spring constant was determined with the thermal tune mode of the Dimensions Icon AFM, and the deflection sensitivity was calibrated on sapphire. The cross section composite was prepared so that the fibers were embedded in polylactic acid (PLA) by melting the PLA at 250 °C using Krüss High-temperature dosing unit (DS4241). Fibers of cellulose with diameter 19.8 µm, cellulose/xylan 15.2 µm, cellulose/lignin 17.5 µm, and cellulose/lignin/xylan 18.6 µm were used for embedding. A cross section was cut with a razor blade and polished using Leica Ultracut R and a diamond blade (ultra AFM, Diatome).

## Electronic supplementary material

Below is the link to the electronic supplementary material.

Supplementary material 1 (PDF 746 kb)

## References

[CR1] Adanur S (1995). Wellington Sears handbook of industrial textiles.

[CR2] Adusumalli R, Keckes J, Martinschitz KJ, Boesecke P, Weber H, Röder T, Sixta H, Gindl W (2009). Comparison of molecular orientation and mechanical properties of Lyocell fibre tow and staple fibres. Cellulose.

[CR3] Alekhina M, Mikkonen K, Alén R, Tenkanen M, Sixta H (2014). Carboxymethylation of alkali extracted xylan for preparation of bio-based packaging films. Carbohydr Polym.

[CR4] Asaadi S, Hummel M, Hellsten S, Härkäsalmi T, Ma Y, Michud A, Sixta H (2016). Renewable high-performance fibers from the chemical recycling of cotton waste utilizing an ionic liquid. Chemsuschem.

[CR5] Blatazar-y-Jimenez A, Bismarck A (2007). Wetting behavior, moisture up-take and electrokinetic properties of lignocellulosic fibres. Cellulose.

[CR6] Chaker A, Alila S, Mutje P, Rei Vilar M, Boufi S (2013). Key role of hemicellulose content and the cell morphology on the nanofibrillation effectiveness of cellulose pulps. Cellulose.

[CR7] Chanzy H, Paillet M, Hagege R (1990). Spinning of cellulose from N-methyl morpholine N-oxide in the presence of additives. Polymer.

[CR8] Colson J, Andorfer L, Nypelö T, Lütkemeier B, Stöckel F, Konnerth J (2017). Comparison of silicon and OH-modified AFM tips for adhesion force analysis on functionalised surfaces and natural polymers. Colloids Surf. A.

[CR9] Fink HP, Weigel P, Purz HJ, Ganster J (2001). Structure formation of regenerated cellulose materials from NMMO-solutions. Prog Polym Sci.

[CR10] Gindl-Altmutter W, Obersriebnig M, Veigel S, Liebner F (2014). Compatibility between cellulose and hydrophobic polymer provided by microfibrillated lignocellulose. Chemsuschem.

[CR11] Good RJ (1993) Contact angle, wetting and adhesion: a critical review. In: Contact angle, wettability and adhesion. Festschrift in Honor of Professor Robert J. Good. Utrecht, pp. 3–36

[CR12] Good RJ, van Oss CJ, Schrader ME, Loeb GI (1992). The modern theory of contact angles and the hydrogen bond components of surface energies. Modern approaches to wettability.

[CR13] Heng JYY, Pearse DF, Thielmann F, Lampke T, Bismarck A (2007). Methods to determine surface energies of natural fibers: a review. Compos Interfaces.

[CR14] Hodgson KT, Berg JC (1988). Dynamic wettability properties of single wood pulp fibers and their relationship to absorbency. Wood Fiber Sci.

[CR15] Hult EL, Larsson PT, Iversen T (2001). Cellulose fibril aggregation—an inherent property of Kraft pulps. Polymer.

[CR16] Hummel M, Michud A, Tanttu M, Asaadi S, Ma Y, Hauru LK, Parviainen A, King AW, Kilpeläinen I, Sixta H, Rojas O (2015). Ionic liquids for the production of man-made cellulosic fibres: opportunities and challenges. Cellulose chemistry and properties: fibres, nanocelluloses and advanced materials.

[CR17] Janson J (1970). Calculation of the polysaccharide composition of wood and pulp. Pap Puu.

[CR18] Kisonen V, Valtakari D, Eklund P, Seppälä J, Tenkanen M, Willför S (2015). Composite films of nanofibrillated cellulose and O-acetyl galactoglucomannan (GGM) coated with succinic esters of GGM showing potential as barrier material in food packaging. J Mater Sci.

[CR19] Kosan B, Michels C, Meister F (2008). Dissolution and forming of cellulose with ionic liquids. Cellulose.

[CR20] Lenz J, Schurz J, Wrentschur E (1994). On the elongation mechanism of regenerated cellulose fibres. Holzforschung.

[CR21] Ma Y, Asaadi S, Johansson L-S, Ahvenainen P, Reza M, Alekhina M, Rautkari L, Michud A, Hauru L, Hummel M, Sixta H (2015). High-strength composite fibers from cellulose-lignin blends regenerated from ionic liquid solution. Chemsuschem.

[CR22] Ma Y, Hummel M, Määttänen M, Särkilahti A, Harlin A, Sixta H (2016). Upcycling of waste paper and cardboard to textiles. Green Chem.

[CR23] Michud A, Tanttu M, Asaadi S, Ma Y, Netti E, Kääriäinen P, Persson A, Berntsson A, Hummel M, Sixta H (2016). Ioncell-F: ionic liquid-based cellulosic textile fibers as an alternative to viscose and lyocell. Textile Res J.

[CR24] Miller B, Young RA (1975). Methodology for studying the wettability of filaments. Textile Res J.

[CR25] Miller B, Penn LS, Hedvat S (1983). Wetting force measurements on single fibers. Colloids Surf.

[CR26] Nypelö T, Laine C, Colson J, Henniges U, Tammelin T (2017). Submicron hierarchy of cellulose nanofibril films with etherified. Carbohydr Polym.

[CR27] Parviainen A, Wahlström R, Liimatainen U, Liitiä T, Rovio S, Helminen J, Hyväkkö U, King A, Suurnäkki A, Kilpeläinen I (2015). Sustainability of cellulose dissolution and regeneration in 1, 5-diazabicyclo [4.3. 0] non-5-enium acetate: a batch simulation of the IONCELL-F process. RSC Adv.

[CR28] Pietak A, Korte S, Tan E, Downard A, Staiger MP (2007). Atomic force microscopy characterization of the surface wettability of natural fibres. Appl Surf Sci.

[CR29] Rojo E, Peresin MS, Sampson WW, Hoeger IC, Vartiainen J, Laine J, Rojas OJ (2015). Comprehensive elucidation of the effect of residual lignin on the physical, barrier, mechanical and surface properties of nanocellulose films. Green Chem.

[CR30] Schild G, Liftinger E (2014). Xylan enriched viscose fibers. Cellulose.

[CR31] Singh S, Murthy Z (2017). Study of cellulosic fibres morphological features and their modifications using hemicelluloses. Cellulose.

[CR32] Sixta H, Schelosky N, Milacher W, Baldinger T, Röder T (2001) Characterization of alkali-soluble pulp fractions by chromatography. In: Proceedings of 11th international symposium on wood and pulping chemistry, 11–14 June, Nice

[CR33] Sixta H, Michud A, Hauru L, Asaadi S, Ma Y, King A, Kilpeläinen I, Hummel M (2015). Ioncell-F: a high-strength regenerated cellulose fibre. Nord Pulp Paper Res J.

[CR34] Stuart T, McCall RD, Sharma HS, Lyons G (2015). Modelling of wicking and moisture interactions of flax and viscose fibres. Carbohydr Polym.

[CR35] Sun N, Li W, Stoner B, Jiang X, Lu X, Rogers R (2011). Composite fibers spun directly from solutions of raw lignocellulosic biomass dissolved in ionic liquids. Green Chem.

[CR36] Sundberg A, Sundberg K, Lillandt C, Holmbom B (1996). Determination of hemicelluloses and pectins in wood and pulp fibres by acid methanolysis and gas chromatography. Nord Pulp Paper Res J.

[CR37] Thunga M, Chen K, Grewell D, Kessler M (2014). Bio-renewable precursor fibers from lignin/polylactide blends for conversion to carbon fibers. Carbon.

[CR38] van Oss CJ, Chaudhury MK, Good R (1988). Interfacial Lifschitz-van der Waals and polar interactions in macroscopic systems. J Chem Rev.

[CR39] Yang Q, Pan X (2015). Correlation between lignin physicochemical properties and inhibition to enzymatic hydrolysis of cellulose. Biotechnol Bioeng.

