# Supplementary material for: Conversion of wood-biopolymers into macrofibers with tunable surface energy via dry-jet wet-spinning
Source: Cellulose (Lond). 2018 Jun 19;25(9):5297–307. doi: 10.1007/s10570-018-1902-4 (PMC6105199; doi:10.1007/s10570-018-1902-4)
Supplement: Supplementary file 1 — Supplementary material 1 (PDF 746 kb) [file 10570_2018_1902_MOESM1_ESM.pdf]

Online Resource

Conversion of wood-biopolymers into macrofibers with tunable surface energy via dry-jet wet-spinning

Tiina Nypelö[a,b]*, Shirin Asaadi[c], Günther Kneidinger[b], Herbert Sixta[c], Johannes Konnerth[b]*

[a] Division of Applied Chemistry, Department of Chemistry and Chemical Engineering, Chalmers University of Technology, Gothenburg, Sweden.

[b] Institute of Wood Technology and Renewable Materials, Department of Material Sciences and Process Engineering, University of Natural Resources and Life Sciences, Vienna, Austria.

[c] Department of Bioproducts and Biosystems, Aalto University, Espoo, Finland.

*corresponding authors

Fiber diameter analysis

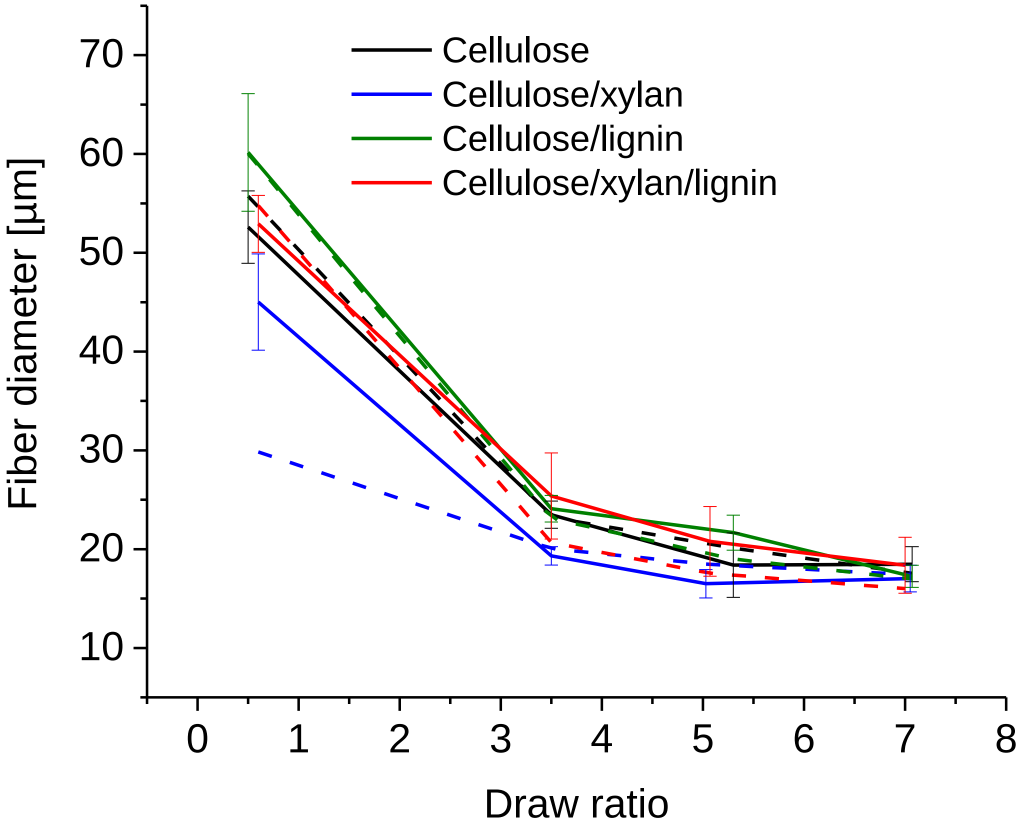

Figure S1. Fiber diameter determined by light microscopy (solid lines) and from vibroscope measurements (dashed lines) after applying a correction factor 0.4 for cellulose and cellulose/xylan fibers, 0.5 for cellulose/lignin fibers and 0.45 for cellulose/xylan/lignin fibers.

Contact angle and swelling of fibers with various liquids

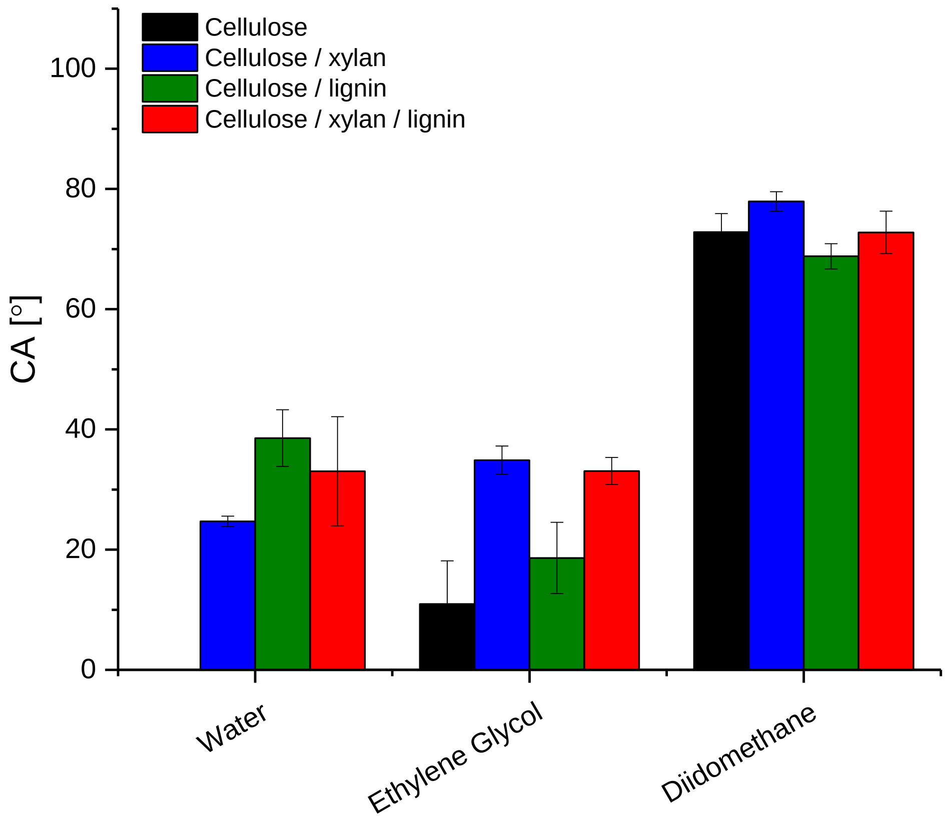

Figure S2. Contact angle (CA) of water, ethylene glycol, glycerol and diidomethane with the cellulose, cellulose/xylan, cellulose/lignin and cellulose/xylan/lignin fibers determined by Wilhelmy plate method.

Observation of effect of immersion to water on fiber structure

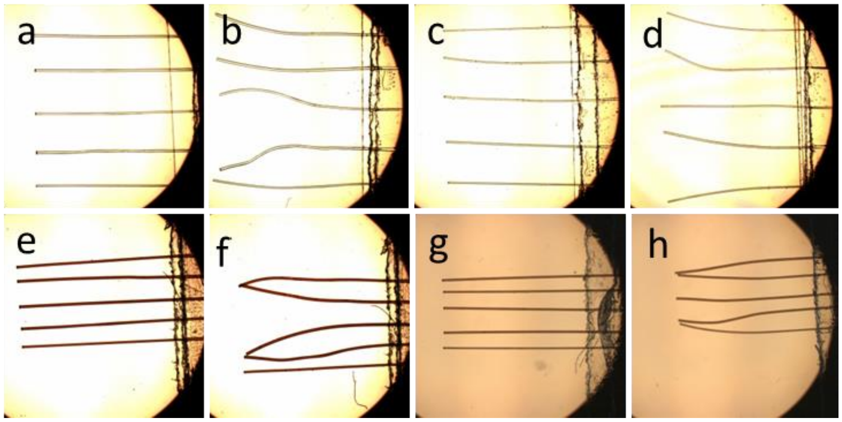

Figure S3. Light microscope images of a) cellulose fiber before, b) cellulose fiber after, c) cellulose/xylan fiber before, d) cellulose/xylan fiber after, e) cellulose/lignin fiber before, f) cellulose/lignin fiber after, g) cellulose/xylan/lignin fiber before, h) cellulose/xylan/lignin fiber after immersion to water for 2 minutes.

Table S1. Fiber cross sectional area change when exposed to diidomethane or ethylene glycol, estimated percental change in total surface energy (SEtot) using contact angle value adjusted with change in wetted length caused by swelling, and adhesion force of the cross section area recorded with AFM adhesion force mapping.

Fiber	Swelling in diidomethane	Swelling in ethylene glycol	*$SEtot_{change}$	Adhesion force (nN)
Cellulose	1.27%±1.89	1.81%±6.54	0.15%	41.7
Cellulose/xylan	-6.90%±4.66	2.14%±1.88	0.64%	47.4
Cellulose/lignin	0.79%±2.91	0.76%±2.55	5.09%	35.3
Cellulose/xylan/lignin	-1.00%±2.98	0.43%±2.13	0.14%	43.7

*Determined using program Surface Energy V1.xls, Copyright © 2007 By Gerhard Sinn.

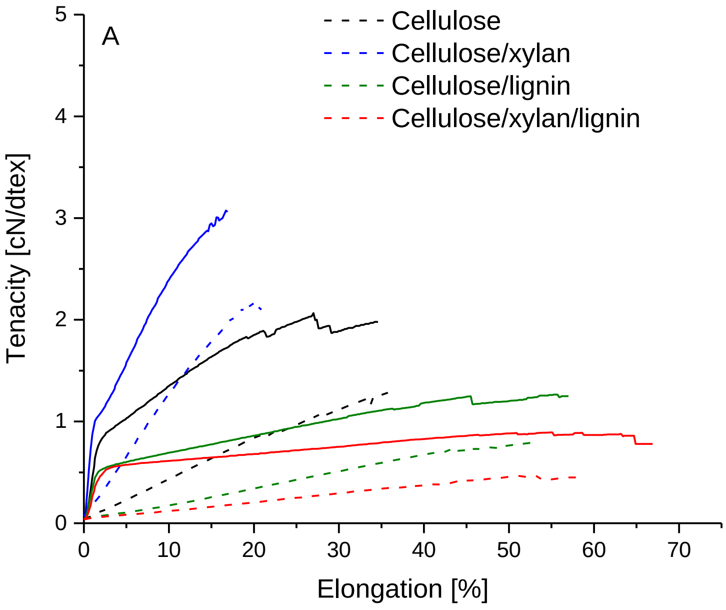
A
Cellulose
Cellulose/xylan
Cellulose/lignin
Cellulose/xylan/lignin
Tenacity [cN/dtex]
Elongation [%]
0
1
2
3
4
5
0
10
20
30
40
50
60
70

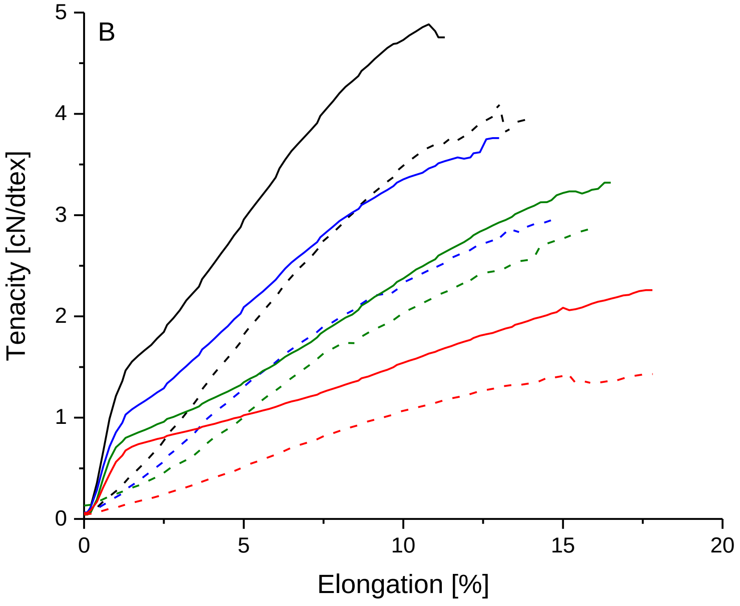
B
Tenacity [cN/dtex]
Elongation [%]
0
1
2
3
4
5
0
5
10
15
20

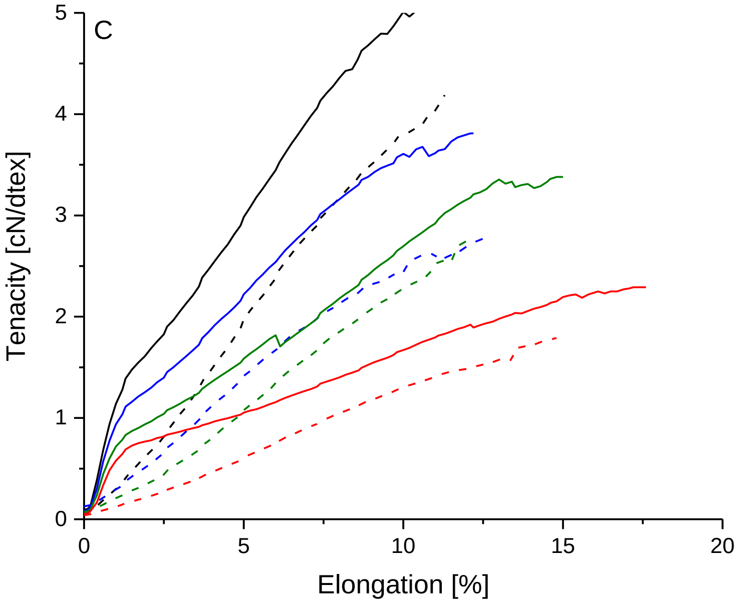

Figure S4. Strength characteristics of the fibers produced with draw ratio of a) 0.5, b) 3, c) 5 and d) 7. The dashed lines represent the strength of wet fibers and the solid line that of conditioned fibers.

Rheological measurements

The rheological characteristics of the spinning solutions under shear stresses were measured. The visco-elastic behavior was studied by means of an Anton Paar MCR 300 rheometer with parallel plate geometry (25 mm plate diameter, 1 mm gap size).

The dynamic frequency sweep was performed with a strain of 0.5% within the angular frequency range of 0.1-100 s-1 at various temperatures from 60 to 85 °C and complex viscosity, storage and loss moduli were recorded. The zero shear viscosity was determined by fitting the complex viscosity data with the three-parameter Cross viscosity model assuming that the Cox-Merz rule was valid.(Coz&Merz 1958; Lu et a. 2012; Sammons et al. 2008)

References

Cox W, Merz E (1958) Understanding rheology of thermoplastic polymers. ASTM Spec. Techn. Publ.247, 178-188.

Lu F, Cheng B, Song J, Liang Y (2012) Rheological characterization of concentrated cellulose solutions in 1-allyl-3-methylimidazolium chloride. J. Appl. Polym. Sci. 124, 3419-3425.

Sammons R, Collier J, Rials T, Petrovan S (2008) Rheology of 1-butyl-3-methylimidazolium chloride cellulose solutions. I. Shear rheology. J. Appl. Polym. Sci. 110, 1175-1181.
